# Optimization of Nile Tilapia Artificial Breeding Using Human Chorionic Gonadotropin (hCG) Hormone

**DOI:** 10.3390/mps8030057

**Published:** 2025-06-02

**Authors:** Golam Rbbani, Prabhugouda Siriyappagouder, Riaz Murshed, Rajesh Joshi, Artem Nedoluzhko, Jorge Galindo-Villegas, Jorge M. O. Fernandes

**Affiliations:** 1 Faculty of Fisheries and Bioscience, Nord University, 8026 Bodø, Norway; baurumon@gmail.com (G.R.); prabhugouda.siriyappagouder@nord.no (P.S.); riaz060707@gmail.com (R.M.); jorge.galindo-villegas@nord.no (J.G.-V.); 2 GenoMar Genetics, 0252 Oslo, Norway; rajesh.joshi@genomar.com; 3 Paleogenomics Laboratory, European University at Saint Petersburg, St. Petersburg 191187, Russia; anedoluzhko@eu.spb.ru; 4 Institute of Marine Sciences (ICM-CSIC), 08003 Barcelona, Spain

**Keywords:** Nile tilapia, human chorionic gonadotropin (hCG), reproduction, ovulation, aquaculture

## Abstract

Nile tilapia (*Oreochromis niloticus*) is the most widely farmed tilapia species globally, making it one of the most important aquaculture species. To meet increasing demand, hatcheries occasionally use artificial breeding techniques such as hormonal induction to synchronize breeding. Despite the common use of human chorionic gonadotropin (hCG) in fish breeding, no detailed protocol has been established specifically for Nile tilapia. The objective of this study is to establish an effective hCG-induced artificial breeding protocol for gene editing and aquaculture production, optimizing fertilization, hatching, and survival rates. We employed a single intramuscular injection of 2 IU/g hCG to induce ovulation. The protocol achieved an average fertilization rate of 88.3% and a larval survival rate of 90.5%, demonstrating its potential for obtaining high-quality embryos for functional studies and enhancing reproductive performance on a commercial scale.

## 1. Introduction

Nile tilapia (*Oreochromis niloticus*) is one of the most important freshwater fish species cultured globally and contributes significantly to the global aquaculture industry. This species alone accounts for approximately 5 million metric tons of annual production, and with increasing demand, global production is projected to reach 7.3 million tons by 2030 [1]. Several factors contribute to the choice of Nile tilapia in aquaculture, including their large size, high growth rates, short lifecycle, prolific breeding attributes, low protein requirements, and omnivorous feeding. The relatively low cost of production compared to other aquaculture species makes Nile tilapia a cost-effective option for fish farmers. In addition to commercial production, Nile tilapia also serves as a valuable model for studying growth and reproductive biology due to its rapid growth and shorter time to reach adulthood compared to many other species [2,3,4]. They can thrive in a wide range of environmental conditions, including varying temperatures, osmotic pressures, alkalinity levels, and low dissolved oxygen concentrations, making them highly adaptable to diverse farming environments [5,6].

The reproduction of Nile tilapia in captivity can be controlled through environmental manipulations, such as adjustments to photoperiod, temperature, or spawning substrate [2,7,8]. Under suitable conditions, Nile tilapia can reach sexual maturity as early as 3 to 4 months after hatching, enabling rapid population growth. Female Nile tilapia are capable of producing between 75 and 1000 offspring every 14 to 22 days of the spawning cycle [9,10]. Achieving successful captive production of Nile tilapia requires proficiency in advanced breeding techniques, ensuring high-quality larvae in sufficient quantities to meet aquaculture demands. Producers with expertise in Nile tilapia breeding methods are increasingly adopting advanced systems to maximize fry production. In certain cases, exogenous hormones are employed to stimulate ovulation in hatchery fish, providing better control of fertilization and enhancing the overall efficiency of the breeding process [2,11].

Ovulation and spermiation in fish can be induced by several varieties of natural and synthetic hormones that are commercially available. For example, human chorionic gonadotropin (hCG), pituitary homogenate, Ovaprim (analogue of salmon gonadotropin-releasing hormone (GnRH) and domperidone as a dopamine receptor antagonist) and Ovopel (mammalian GnRH analogue and metoclopramide as a dopamine receptor antagonist) are commonly used for artificial breeding [12,13,14,15]. Hormonal induction protocols are widely utilized in fish breeding to facilitate the collection of gametes for chromosome manipulation, reproductive biology research, and selective breeding programs. The use of hormonal induction is also very important for the breeding and management of fishes that migrate during their spawning season, such as Neotropical reofilic fish, which normally do not spawn naturally in captivity. Injection with carp pituitary extract has been successfully used to promote spawning in five such species of commercial importance (*Piaractus mesopotamicus*, *Brycon orbignyanus*, *Prochilodus lineatus*, *Salminus brasiliensis*, and *Leporinus macrocephalus*) [16], as well as in *Astyanax altiparanae*, which is proposed as a model fish for the Neotropical region [17].

The effectiveness of hormonal treatments depends on factors such as dosage, the targeted species, and the maturation stage of the fish. Among the commonly used hormones, hCG is one of the most popular and effective solutions for stimulating ovulation in female broodfish. Despite the effectiveness of hCG in stimulating ovulation, only three studies have specifically documented its use in Nile tilapia breeding, focusing primarily on its efficacy rather than its use in commercial aquaculture settings [12,13,18]. Developing a detailed protocol could significantly aid fish farms in utilizing hCG effectively on a commercial scale while minimizing its residual hormonal effects. To address this gap, we present a detailed protocol of the method that we used in a recent study for synchronizing final oocyte maturation using hCG in the artificial reproduction of Nile tilapia [3]. In addition to its relevance for functional studies in the laboratory (e.g., for gene editing), hCG-induced breeding methods may find application in the aquaculture industry, improving breeding efficiency and production outcomes.

## 2. Experimental Design

### Materials

- Mature male and mature female Nile tilapia (*Oreochromis niloticus*);
- Human chorionic gonadotropin (Merck, Darmstadt, Germany);
- NaCl (sodium chloride 98.80%) (Maldon, Essex, UK);
- Nitrate-Nitrite (0–40 mg/L NO_3_^−^-N, 0.015–0.60 mg/L NO_2_^−^-N) and ammonia test kit (0–2.4 mg/L NH_3_-N) (product numbers: 1408100 and 2428700, Hach, Loveland, CO, USA);
- 100 L plastic buckets;
- Heater (EHEIM thermocontrol 150 and 300 Watt, cat-4011708011713 and 4011708011744, Eheim GmbH & Co., Deizisau, Germany);
- Aerator (TetraTec APS 120-400, 400 L/h 4.5 watts, Tetra GmbH, Melle, Germany);
- Syringe (0.3 mL, Beckton-Dickinson, Oslo, Norway);
- Needle (Gauge 22, WVR, Radnor, PA, USA);
- Borosilicate glass beaker (500 mL);
- Aquarium tanks (500 L);
- Fine mesh net (7.5 cm)
- Lamp (Unilite SLR-3000, Worcestershire, UK);
- Timer (Bachmann, Bremen, Germany);
- Egg rocker (5 L capacity) (item number 1407, FIAP GmbH, Ursensollen, Germany);
- Air pump (Eheim Compacton 600 Pump, Deizisau, Germany);
- EBI temperature probe (item no. 620-1860P, WVR, Radnor, PA, USA);
- pH meter (item no. 665-0499P, WVR, Radnor, PA, USA);
- Oxygen meter (H05: Polaris C, Oxyguard, Farum, Denmark);
- Leica MZ16 FA dissecting stereomicroscope (Leica Microsystems GmbH, Wetzlar, Germany).

## 3. Procedure

### 3.1. Selection of Female Tilapia

**Figure 1:**
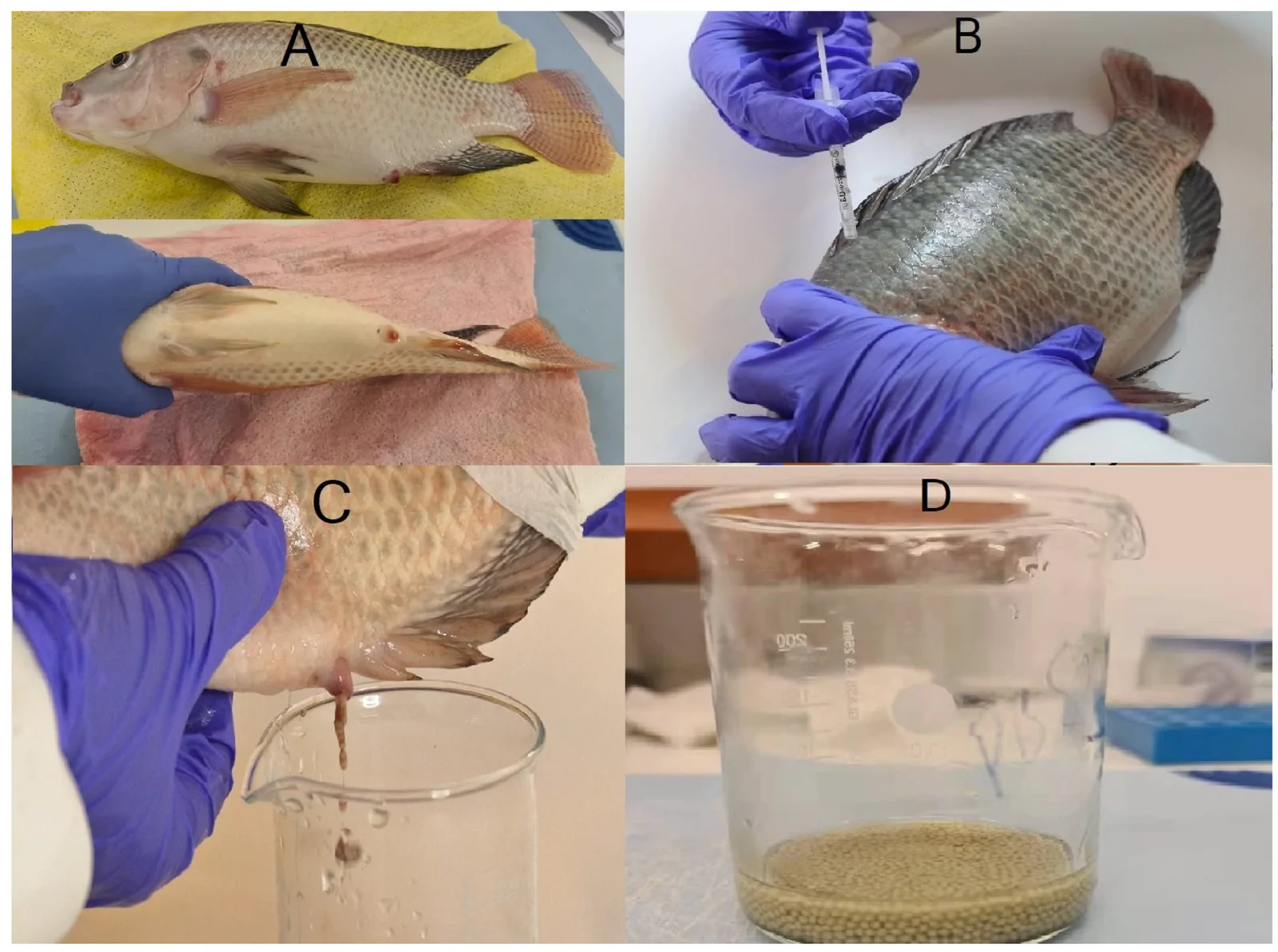
Illustration of the hormone-induced spawning protocol in Nile tilapia (*Oreochromis niloticus*). (**A**) Sexually mature female with prominent reddish genital papilla and pinkish fin color. (**B**) Intramuscular injection of hCG below the dorsal fin. (**C**) Manual egg extrusion was performed by applying gentle pressure along the abdominal cavity toward the genital pore. (**D**) Eggs exhibiting optimal golden-yellow coloration and uniform size distribution.

Select one mature female Nile tilapia by looking for the following secondary sexual characteristics: reddish genital papilla, pinkish fin color, and a swollen abdomen (as shown in Figure 1A).

### 3.2. Hormone Induction

Administer an intramuscular injection of hCG below the dorsal fin to the selected female at a dosage of 2 IU/g body weight, based on our preliminary trials (see Figure 1B).

### 3.3. Post-Injection Care

Place the injected female in a 100 L bucket, 2/3 full of water; with continuous aeration, maintain the fish there for 24 h. Keep the water temperature stable at 28 °C using an EHEIM thermocontrol 150 heater (cat-4011708011713, Eheim GmbH & Co., Deizisau, Germany).

### 3.4. Selection of Male Tilapia

Collect male tilapia that are ready to release sperm by identifying the following secondary sexual characteristics: pointed dorsal and anal fins and a noticeable reddish coloration on the body.

### 3.5. Egg Collection

After 24 h post-injection, gently massage the female’s abdomen to collect the eggs into a clean beaker (Figure 1C).

### 3.6. Sperm Collection and Fertilization

Immediately strip the male and collect the sperm directly into the beaker containing eggs. Add water (approximately 2 mL) to activate the sperm, and gently shake (by hand or using a feather) the beaker continuously for 8–10 min to ensure the mixing of sperm and eggs (Figure 1D).

### 3.7. Washing Fertilized Eggs

After fertilization, rinse the fertilized eggs 5–6 times with system water (100 mL) to remove debris and redundant sperm.

### 3.8. Incubation

**Figure 2:**
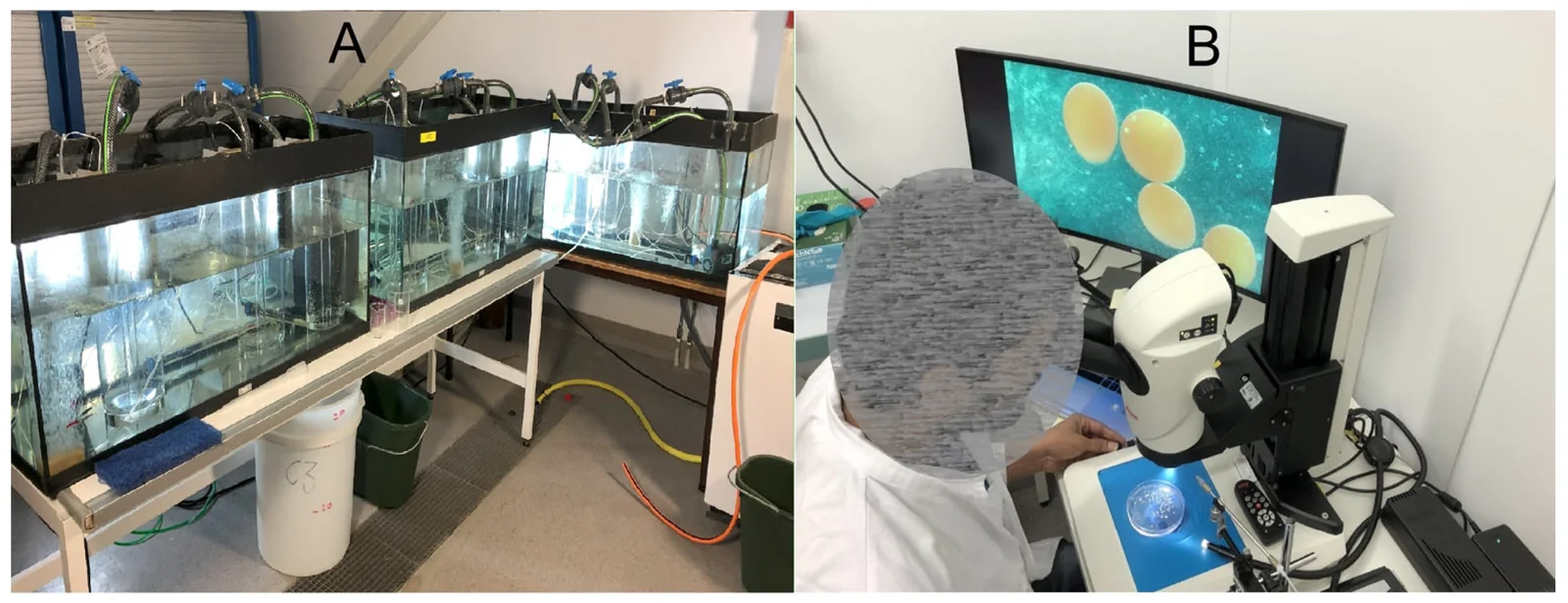
Incubation setup (**A**) and fertilized egg monitoring (**B**). Fertilized eggs are incubated in egg rockers placed in tanks with continuous water flow, maintained using a water pump. Regular monitoring of the developing embryos is performed using a Leica MZ16 FA dissecting stereomicroscope (Leica Microsystems GmbH, Wetzlar, Germany), precisely mounting them on a Petri dish and securing them in place using 3% methylcellulose (Sigma-Aldrich, St. Louis, MO, USA). Monitoring helps in assessing the viability of the embryos during the incubation period.

Transfer the fertilized eggs to three different tanks (500 L) containing three egg rockers (5 L) each. Each egg rocker contained 230 fertilized eggs, ensuring appropriate temperature and aeration conditions were maintained (see Figure 2A). Tank temperature (24 °C, 28 °C and 32 °C) was maintained using the EHEIM thermocontrol 300 (cat-4011708011744, Eheim GmbH & co., Deizisau, Germany).

### 3.9. Water Quality Parameters

Dissolved oxygen was determined with a Polaris C meter (Oxyguard, Farum, Denmark), and pH, ammonia, nitrite, and nitrate were periodically monitored during the entire trial using appropriate measuring kits (Supplementary Table S1).

### 3.10. Photoperiod

Maintain fertilized embryos under an artificial photoperiod of 13 h light and 11 h dark (13 h:11 h) using lamps (3000 lm, Unilite SLR-3000, Redditch, UK) controlled by a timer.

### 3.11. Monitoring the Fertilized Eggs

Monitor the fertilized eggs every 6 h for 16 days until the larvae absorb the yolk sac (Figure 2B).

### 3.12. Data Collection

Calculate fertilization rate, hatching rate, and larval survival rate as development progresses. Fertilization rate was determined as the percentage of embryos relative to the total number of eggs, multiplied by 100. The hatching rate was calculated as the percentage of embryos that successfully hatched at 96, 128, and 200 h post-fertilization at 32, 28, and 24 °C, respectively. The survival rate was based on the number of larvae that survived 3 days after hatching. These metrics were used to assess the effectiveness of the incubation process and the overall health and viability of the developing embryos.

## 4. Results and Discussion

In this study, fertilized eggs were incubated in a large tank using egg rockers and replicated in triplicate at each temperature until yolk sac absorption [3]. Continuous water flow was maintained in the tanks using a pump to ensure that the eggs remained in constant motion, preventing them from settling at the bottom (Figure 2A). This setup closely mimics the natural mouth incubation process in Nile tilapia.

Table 1 shows the fertilization, hatching, and survival rates in the breeding trials. The fertilization rates were assessed 1 day post-fertilization, and hatching rates were recorded 3 days post-fertilization. Across all temperature groups, the fertilization rates were consistently high, ranging from approximately 82.6% to 91.3%. The hatching rates showed some variation across different temperatures. The highest hatching rate was observed at 24 °C in Replicate 3 (80.5%). Finally, the survival rate of larvae post-hatching remained relatively high across all temperature conditions. The highest survival rate was observed at 32 °C in Replicate 2 (94.6%), while the lowest was at 24 °C in Replicate 3 (88.9%).

These results are in line with those of previous studies examining the effects of hormonal treatments on Nile tilapia reproduction. For instance, Fernandes et al. [13], using a large number of fish, achieved a spawning percentage ranging from 53% to 82%. Importantly, the untreated group produced no larvae at the end of the study, underscoring the effectiveness of hormonal treatments. Similarly, El-Gamal and El-Greisy reported comparable fertilization rates but used a hormone dose 25 times higher than the dose applied in this study [19]. These findings highlight the efficiency and practicality of this protocol, particularly in achieving high reproductive outcomes with a significantly lower hormonal dosage.

Egg spawning of Nile tilapia in farms often poses challenges due to asynchronous influences of various factors, including temperature, photoperiod, oxygen availability, and rainfall [20,21,22]. Temperature proves particularly influential, as Nile tilapia experience a wide range of temperatures due to strong seasonal thermal variations, with natural ranges spanning 22 °C to 34 °C [23,24]. These variations are encountered in diverse aquatic ecosystems, such as rivers, lakes with consistently cooler temperatures, and hydrothermal hot springs. The selected incubation temperatures in this study (24 °C, 28 °C, and 32 °C) were chosen to reflect this natural thermal adaptability and to assess the potential impacts of environmental temperature variations on reproductive success. Comparative analysis of the results across temperatures and developmental stages—fertilization, hatching, and larval survival—indicates that 28 °C offers the most balanced outcomes. This underscores the importance of maintaining optimal thermal conditions to improve reproductive efficiency and aquaculture practices.

Furthermore, the hormonal induction protocol facilitates the collection of fully mature eggs, thus ensuring efficient fertilization, which is crucial for the success of gene-editing techniques such as CRISPR/Cas9. The resulting embryos provide a reliable biological platform for microinjecting genetic material, enabling enhanced precision and consistency in genetic manipulations [25,26,27]. In contrast, traditional methods of egg collection, which rely on observing females and harvesting eggs during spawning in aquariums, often result in variability due to asynchronous oocyte maturation and inconsistent developmental stages of embryos for gene editing. Such inconsistencies can lead to lower fertilization rates, impede gene-editing efficiency, and reduce the reproducibility of experimental outcomes. By addressing these limitations, this protocol enables the collection of high-quality eggs with uniform maturity, thereby enhancing the success of gene-editing experiments and promoting the broader application of genetic tools in aquaculture and scientific research.

To achieve these results, it is important to focus on several key points. These are as follows:

1. Breeders should be maintained in a controlled wet lab environment for proper evaluation, ensuring the selection of superior females. Housing selected males and females in separate aquarium systems facilitates female maturation for spawning and allows more effective monitoring and management.
2. Throughout the incubation process, we carefully monitored the eggs, removing any dead embryos to prevent deterioration of water quality.
3. Sperm was stripped from the male and directly added to the eggs to ensure its viability. This immediate mixing helps maximize the chances of successful fertilization.
4. It is essential to keep the eggs in motion at the bottom of the egg rockers to ensure successful development.
5. The water in the tanks was changed once daily and replaced (75%) with freshwater manually using a bucket.
6. When calculating the concentration of hormones used during breeding, particular attention should be given, as even slight variations in hormone dosage can have a significant impact on fertilization rates and larval survival. Proper dosing is crucial to ensuring successful reproduction and optimal outcomes in the breeding process.

## Figures and Tables

**Table 1:** Breeding data collected throughout the trial.

Group	Replicate	Total Eggs	Dead Eggs	Fertilized Eggs	Fertilization Rate (%)	Hatch-Out Larvae	Hatching Rate (%)	Total Surviving Larvae	Survival Rate (%)
32 °C	Rep 1	230	26	204	88.7	135	66.2	113	83.7
	Rep 2	230	24	206	89.6	129	62.6	122	94.6
	Rep 3	230	20	210	91.3	127	60.5	116	91.3
28 °C	Rep 1	230	22	208	90.4	140	67.3	128	91.4
	Rep 2	230	32	198	86.1	145	73.2	131	90.3
	Rep 3	230	25	205	89.1	148	72.2	133	89.9
24 °C	Rep 1	230	22	208	90.4	147	70.7	137	93.2
	Rep 2	230	31	199	86.5	151	75.9	138	91.4
	Rep 3	230	40	190	82.6	153	80.5	136	88.9

## Data Availability

The original contributions presented in this study are included in the article/Supplementary Material. Further inquiries can be directed to the corresponding authors.

## Supplementary Materials

The following supporting information can be downloaded at: https://www.mdpi.com/article/10.3390/mps8030057/s1, Table S1: Water quality parameters (dissolved oxygen, pH, salinity, nitrates, nitrites and ammonia) throughout the experimental period.
