# Supplementary material for: Optimization of Nile Tilapia Artificial Breeding Using Human Chorionic Gonadotropin (hCG) Hormone
Source: Methods Protoc. 2025 Jun 2;8(3):57. doi: 10.3390/mps8030057 (PMC12195898; doi:10.3390/mps8030057)
Supplement: Supplementary file 1 [file mps-08-00057-s001.zip › mps-3353860-supplementary.pdf]

Supplementary Table S1. Water quality parameters (dissolved oxygen, pH, salinity, nitrates, nitrites and ammonia) throughout the experimental period.

Water quality measurement									
Day		Day 0	Day 1	Day 2	Day 3	Day 4	Day 5	Day 6	Day 7
Photoperiod		13:11	13:11	13:11	13:11	13:11	13:11	13:11	13:11
Temperature group		6.8	6.65	6.8	6.8				
Dissolved O2 (mg/L)	32 ˚C								
	28 ˚C	7.2	7.16	7.5	7.2	6.4			
	24 ˚C	7.7	7.62	7.7	7.6	7.7	7.5	7.7	7.6
pH	32 ˚C	7.53	7.95	7.96	7.99				
	28 ˚C	7.66	8.01	7.88	7.92	8.07			
	24 ˚C	7.88	7.85	7.92	8.01	8.05	7.93	7.95	7.98
Salinity (gm/L)	32 ˚C	2.0-2.5	2.0-2.5	2.0-2.5	2.0-2.5				
	28 ˚C	2.0-2.5	2.0-2.5	2.0-2.5	2.0-2.5	2.0-2.5	2.0-2.5	2.0-2.5	
	24 ˚C	2.0-2.5	2.0-2.5	2.0-2.5	2.0-2.5	2.0-2.5	2.0-2.5	2.0-2.5	2.0-2.5
Nitrates*	32 ˚C	0			0				0
	28 ˚C	0			0				0
	24 ˚C	0			0				0
Nitrites*	32 ˚C	0			0				0
	28 ˚C	0			0				0
	24 ˚C	0			0				0
Ammonia*	32 ˚C	0.018			0.4				0
	28 ˚C	0.011			0.42				0
	24 ˚C	0.021			0.42				0.024

* Nitrate, nitrites and ammonia were analyzed thrice during the experimental period since we changed half of the tank water daily.
